# The urgency to expand the antiviral indications of general chronic hepatitis B patients

**DOI:** 10.3389/fmed.2023.1165891

**Published:** 2023-05-19

**Authors:** Ping Fan, Lan-Qing Li, En-Qiang Chen

**Affiliations:** ^1^Department of Pharmacy, West China Hospital, Sichuan University, Chengdu, China; ^2^Center of Infectious Diseases, West China Hospital, Sichuan University, Chengdu, China

**Keywords:** chronic hepatitis B, antiviral indication, normal ALT, age, general population

## Abstract

In recent years, liver experts have conducted in-depth discussions on whether it is necessary to expand the indication of antiviral therapy for patients with chronic hepatitis B (CHB). Currently, the guidelines are too strict in treating CHB patients. With the deepening understanding of the natural history of hepatitis B virus infection, there is more and more evidence challenging the view that there is no disease progression and no treatment in the immune tolerance period and inactive period. As the price of antiviral agents for CHB has decreased significantly, the availability of antiviral agents for CHB has been considerably improved. Therefore, expanding the indications for antiviral treatment of CHB is of great significance in achieving the goal of eliminating the public health threat of viral hepatitis by 2030, as the World Health Organization has proposed.

## 1. Introduction

There are approximately 70 million cases of chronic hepatitis B virus (HBV) infection in China at present, accounting for approximately 1/3 of the global number of HBV infections, including 20 ~ 30 million with chronic hepatitis B (CHB) (1). Approximately 300,000 people die of hepatitis B-related cirrhosis and hepatocellular carcinoma (HCC) each year, accounting for 50% of the world’s hepatitis B-related deaths (2).

For CHB patients, timely and effective antiviral therapy can inhibit HBV replication, reduce liver inflammation and necrosis, and block or reverse liver fibrosis and even early cirrhosis, thereby reducing cirrhosis-related complications, hepatocellular carcinoma (HCC), and liver disease-related mortality. Unfortunately, many patients fail to receive antiviral therapy because they do not meet the existing indications of antiviral treatment, which may lead to disease progression or even death. A prospective follow-up study of 369 HBsAg-positive patients in the United States (3) showed that 30 patients developed HCC during an average follow-up period of 7 years, and 37 patients died of non-HCC liver disease-related deaths. Moreover, in all patients, 40% ~ 80% of HCC patients and 30% ~ 73% of non-HCC liver disease-related deaths did not meet the antiviral therapy indications recommended by the guidelines. In another multi-center cohort study from South Korea in 2019 on 3,624 untreated CHB patients, those who did not meet the antiviral therapy indications accounted for 33.5% ~ 64% of HCC deaths (4). These data indicate that many CHB patients who do not meet the existing indications of antiviral therapy are still progressing quietly. If these patients receive antiviral treatment, the risk of death from HCC and non-HCC can be reduced.

In China, the current diagnosis and treatment rates of CHB are not satisfactory. According to data from the China Registry of Hepatitis B, only 2.8 million (10%) CHB patients are receiving the needed treatment (5). How to expand the antiviral therapy population and let more patients who do not meet the existing indications but have a potential risk of disease progression receive antiviral therapy are issues that require attention. Many guidelines and consensus statements on the management of CHB have greatly expanded the indications for antivirus therapy. Due to the different laboratory testing conditions in other places, inadequate recognition of the importance of antiviral treatment by doctors and patients, and the lack of clear recommendations on clinical doubts in the guidelines, clinicians have different opinions on when to start antiviral therapy for chronic hepatitis B. This article will review the significance of expanding the indications of antiviral therapy for CHB and discuss some relevant issues.

## 2. New understanding of the natural history of HBV infection

In the past decades, the natural history of chronic HBV infection has been generally divided into four stages according to the level of HBV DNA and ALT, HBeAg status, and liver inflammation (6): immune tolerance stage (HBeAg-positive chronic HBV infection), immune clearance stage (HBeAg-positive CHB), immune control stage (HBeAg-negative chronic HBV infection), and reactivation stage (HBeAg-negative CHB). However, in actual clinical work, many patients do not fully meet the above stages. Currently, we consider this section of the population to be in an “uncertain period” or “gray zone.” Some scholars have defined the “uncertain period” as the untreated patients with chronic HBV infection who were followed up for one year. However, their HBV DNA and ALT patterns differed from the four traditional stages of chronic HBV infection (7–9). One study classified the immune status of enrolled CHB patients based on the American Association for the Study of Liver Diseases (AASLD) guideline, and approximately 28% (1,322/4759) of patients fell into the “gray zone” (10). In this group of patients, the ALT level may be consistent, but other indicators are inconsistent, or the HBV DNA level may be consistent, but the ALT level does not meet the above stages.

Though the HBV DNA and ALT levels meet the requirements of the natural history of chronic HBV infection, there are still possible significant liver histological changes that do not meet the definition requirements of the natural history of chronic HBV infection. For example, a study on 179 HBeAg-positive patients with high HBV DNA levels and continuous normal ALT in the so-called immune tolerance period showed that the “uncertainty period” of liver inflammation and fibrosis G2/F2 found by liver biopsy accounted for 57.5% ~ 81.8%. One study from China enrolled 181 HBeAg-positive CHB patients with HBV DNA > 10^7^ IU/mL and persistently normal ALT. The results indicated that approximately 33% had evident histological liver injury based on liver biopsy (11). Another study on 327 HBeAg-negative patients with low HBV DNA levels and continuous normal ALT in the so-called inactive period found that the “uncertain period” of liver inflammation and fibrosis G2/F2 found by liver biopsy accounted for 51.6% ~ 74.5% (12). It can be seen that it is not accurate to determine the “uncertain period” only according to whether the HBV DNA and ALT levels conform to the four traditional staging characteristics of chronic HBV infection. If the HBV DNA and ALT levels were consistent with the stage of chronic HBV infection, a liver biopsy should be performed when necessary to avoid missing this group of patients in the “uncertain period.”

This new understanding of the natural history of chronic HBV infection challenges the view that there is no disease progression in the past immune tolerance period and inactive period, and no antiviral treatment is required. A study by Professor Kim et al. in 2018 showed that the risk of HCC and death or liver transplantation in untreated patients with HBV infection in the so-called immune tolerance period was higher than in patients with CHB in the immune active period (13). In fact, for patients in the actual immune tolerance period or the immune control period (inactive HBsAg carrier status), their conditions are relatively stable, and the future development of the disease is also relatively stable (14). However, patients with chronic HBV infection in the “uncertain period” do not seem to conform to the indications of antiviral therapy in the current guidelines (15). Their risk of long-term progression to cirrhosis and liver cancer is much higher than that of patients in the real immune tolerance period or inactive HBsAg carrier status. The latest Expert Opinions on Expanding Antiviral Therapy for CHB pointed out that antiviral therapy is recommended for CHB patients who are in an “uncertain period” without treatment and whose HBV DNA and ALT patterns are difficult to determine after more than one year of follow-up. Of course, some new virus-related biomarkers reported in recent years, such as hepatitis B core-related antigen (HBcrAg), quantification of anti-HBc (qAnti-HBc), and HBV RNA, have good correlations with the transcription and replication levels of cccDNA in the liver. These new indicators can assist in the clinical identification of so-called “uncertain periods” and help evaluate the efficacy of antiviral therapy and disease prognosis. We believe this will help delay or prevent liver fibrosis and cirrhosis in these patients and reduce the risk of HCC and death or liver transplantation.

## 3. Normal serum ALT does not mean that the liver has no inflammation or fibrosis

For a long time, serum ALT has been considered the most direct and economic indicator of liver inflammation. Long-term ALT abnormalities are strongly associated with the risk of developing HCC. The prognosis of HCC patients with ALT <40 IU/L was better than that of the patients with ALT ≥40 IU/L, showing that ALT is inversely correlated with survival time in HCC patients (16). Elevated ALT has also been regarded as an essential indicator of antiviral therapy for patients with non-end-stage liver disease by international guidelines. For patients with ALT within the normal range, if there is no evidence of progressive liver fibrosis, cirrhosis, and liver cancer, follow-up is generally recommended without advocating antiviral treatment. According to the current antivirus treatment standards of the EASL (7), AASLD (8), or APASL (17) guidelines, a multi-center, retrospective Korean cohort study included 3,624 untreated chronic HBV-infected people (4). In this study, 33.5% of patients with HCC did not meet the EASL 2017 treatment standards, 46.0% did not meet the AASLD 2018 treatment standards, and 64.0% did not meet the APASL 2015 treatment standards. In various international guidelines, the definition of ALT threshold in the natural course of chronic HBV is not entirely consistent. For example, EASL guidelines require that the ALT threshold of the immune clearance period be >1× the upper limit of normal (ULN) (7), while AASLD guidelines require that the ALT threshold be >2× the ULN (8). It is not difficult to find from the results of this retrospective cohort study in South Korea that when the ALT threshold of antiviral treatment standard is lower, the proportion of patients with HCC who do not meet the treatment standard is lower (18). Even if the ALT of chronic HBV-infected persons is in the so-called normal range, it does not mean that the liver is not inflamed or that the liver disease is stable. It is well known that the increase in serum ALT is not parallel to the degree of liver inflammation. The peripheral blood ALT level may be normal when liver inflammation is pronounced. In contrast, when the liver inflammation is slight or not noticeable, the peripheral blood ALT level may be very high (19, 20). In addition, the ALT level does not represent the degree of liver fibrosis (for example, in some patients with liver cirrhosis or even liver cancer, the ALT level is usually within the normal range) (21). One study found that, even among CHB patients with persistently normal ALT, approximately 12.9% had significant necroinflammation, and 36.6% had significant fibrosis (22). Moreover, many patients with acute hepatitis have very high serum ALT levels despite mild liver tissue lesions. Therefore, the serum ALT level cannot accurately reflect the progress of the disease. Currently, the clinic cannot decide whether to start antiviral treatment based on whether the ALT level is within the normal range.

Traditionally, the ULN of serum ALT level is believed to be less than 40 U/L. ALT is vulnerable to the interference of alcohol, drugs, testing reagents, and other factors, and the ULN defined in the past cannot wholly exclude these factors (23–25). Therefore, experts and scholars in many countries and regions call for lowering the upper limit of the normal ALT value. For example, AASLD guidelines reduce the normal ALT range of men and women to 35 U/L and 25 U/L, respectively (8). This change may be more conducive to the early diagnosis and treatment of chronic HBV infection. Even though the general population’s upper limit of normal ALT value cannot be reduced, it should at least be considered to reduce the ALT threshold of antiviral indications for chronic HBV-infected people. According to guidelines and consensus opinions, the ALT threshold recommended for starting the treatment of chronic hepatitis B is approximately 30 U/L for men and 19 U/L for women. In addition, studies have investigated the cost-effectiveness of different serum ALT thresholds as the initiation of antiviral therapy. The lower ALT treatment thresholds are closely related to reducing HBV-related complications and deaths. For example, for HBeAg-negative CHB patients with normal ALT and HBV DNA ≥ 2000 IU/mL, ALT >20 U/L is a good independent predictive factor for evaluating significant liver histopathology (12). Early implementation of an expanded antiviral treatment strategy based on adjusting ALT thresholds will help reduce HBV-related complications and deaths (26).

## 4. Untreated patients with hypoviremia also face the risk of disease progression

Hypoviremia (LLV) is a hot topic in the clinical treatment of chronic hepatitis B. The concept of hypovolemia originally came from AIDS research and refers to the number of viruses in patients’ bodies continuously maintained at a low level during antiviral treatment. However, it does not entirely disappear (27). Hypoviremia also exists in patients with chronic HBV infection, which means that the hepatitis B DNA load in plasma is lower than 2000 IU/mL (fluctuation range is 20 ~ 2000 IU/mL) if HBV persists or can be detected occasionally (8). Ordinary PCR reagents cannot detect this situation; only highly sensitive PCR reagents can do so (28). In the past, it was believed that the low HBV DNA load had little impact on the progress and harm of the disease. Therefore, for patients whose HBV DNA is detected but whose viral load does not reach a certain standard, many countries or regions do not recommend antiviral treatment in the guidelines for hepatitis B (6). It has been proved that chronic HBV-infected persons’ clinical outcome (including the incidence of liver cirrhosis and liver cancer) is related to HBV DNA load (29–31). The higher the HBV DNA load, the higher the risk of liver cirrhosis and liver cancer, but a lower viral load does not impact the occurrence of liver cirrhosis and liver cancer (32–36). The level of HBV DNA is not necessarily consistent with the severity of pathological changes in the liver. It is common to see patients with high virus levels in the immune tolerance period but relatively mild pathology in clinical practice and patients with relatively low virus replication in the immune control period but severe liver pathology.

A recent retrospective study in China (12) showed that 114 (51.6%) of 221 so-called “inactive” HBV-infected persons (ALT<40 U/L, HBV DNA < 2000 IU/mL, HBeAg-negative) had liver tissue pathology showing G2 and/or F2, which was not in the actual inactive period but belonged to the uncertain period. For these patients, if highly sensitive PCR reagents are used to detect HBV DNA, it does not rule out that many patients may detect HBV DNA in their peripheral blood. For patients who have never received antiviral therapy, approximately 10% of patients with persistent hypoviremia develop active hepatitis. In addition to causing hepatitis reactivation in patients with chronic HBV infection, hypoviremia may also promote the progression of liver fibrosis and cirrhosis and increase the risk of liver cancer and is not conducive to the survival of patients with liver cancer (32, 36–38). In addition, this hypoviremia may also be associated with HBV drug resistance (39). In fact, more and more experts suggest antiviral treatment for chronic hepatitis B patients with low-level HBV DNA replication if they are accompanied by one of the following risk factors, regardless of whether the patient’s ALT is abnormal (9): patients over 30 years old, patients with cirrhosis or liver cancer in the family, patients with apparent inflammation (G ≥ 2) or fibrosis (*F* ≥ 2) in the liver indicated by non-invasive indicators or liver histological examination, or extrahepatic manifestations related to HBV infection. Although the evidence of high-quality, evidence-based medicine in this area is not sufficient at present, based on the currently available clinical research evidence, doctors’ clinical experience, and patients’ wishes for treatment, antiviral treatment for patients with hypoviremia should be encouraged rather than conservative follow-up observation. While expanding the population screening for HBsAg, we encourage sensitive and accurate quantitative detection of HBV DNA (the lower limit of HBV DNA detection is 10–20 IU/mL) for HBsAg-positive people. Thus, we can find patients with hypoviremia in a timely manner and patients who need treatment as soon as possible.

## 5. Age is critical to initiating antiviral therapy

As we all know, because the immune system of newborns or infants is not yet fully mature, they cannot clear the virus entirely after infection with HBV, and more than 90% will become chronic virus-infected persons (40, 41). After infection with HBV, as long as the immune function of adults is as expected, more than 95% of patients can spontaneously clear the virus, and very few patients become chronically HBV infected (40). Therefore, for most people with chronic HBV infection, their age can better reflect the duration or course of HBV infection. However, as the body’s immune system matures, the virus also mutates under the immune pressure of the body, and this immune tolerance state may be broken at any time. The immune system causes liver inflammation and fibrosis while clearing the virus (41). A study on 253 patients with chronic HBV infection with normal ALT levels showed that 42% of patients over 40 had prominent liver fibrosis compared with only 30% of patients under 40 (42). In patients who had ALT≤40 U/L, aged <35 years, 36–50 years, and > 50 years, the proportion of liver fibrosis stage 2 was 10, 33, and 53%, respectively (43). Another study showed that age was an independent risk factor associated with significant liver fibrosis (44, 45). For chronic HBV-infected persons with normal ALT levels, their age plays a crucial role in deciding whether to start treatment.

AASLD, Sweden, Taiwan, and East Asian expert opinions all recommend that patients in the immune tolerance period can start antiviral treatment when they are over 40 (8, 46–48). The CGH treatment process in the United States and APASL guidelines recommend starting antiviral treatment at the age of >35 years (17, 49), while EASL and India’s guidelines recommend starting antiviral treatment at the age of >30 years for patients with chronic HBV infection (7, 50). In addition, China’s Expert Opinion on Expanding Antiviral Treatment for Chronic Hepatitis B also recommends that people with chronic HBV infection who are older than 30 years old should start antiviral treatment as long as their HBV DNA is positive (9). Professor Koffas and his team found that the immune tolerance period of HBV infection is 20–30 years (51). However, chronic hepatitis B patients in China are mainly infected with HBV through mother-to-child transmission (52). Therefore, most Chinese chronic hepatitis B patients aged >30 years are no longer in the immune tolerance period of chronic HBV infection. There is an imbalance between age and disease progression to cirrhosis or liver cancer. For example, in daily clinical practice, patients older than 30 may remain in the chronic inflammatory stage without cirrhosis or HCC; on the contrary, some patients less than 30 years old may develop cirrhosis or liver cancer. Therefore, chronic HBV-infected persons with detectable HBV DNA in China should start antiviral treatment promptly if they are >30 years old.

## 6. Summary

With the development of therapeutic drugs for CHB, the accumulation of antiviral experience of clinicians, and the improvement of patient awareness of the disease and treatment compliance, it is imperative to expand the indications for the antiviral treatment of chronic hepatitis B (53). What differs from the previous situation is that the price of antiviral drugs has dropped significantly, and the accessibility of antiviral treatment is no longer a problem. Nucleos (t) ide analogues with solid effects and low drug resistance have become the first choice drugs for all newly treated patients with hepatitis B. It is evident that antiviral treatment itself is not complex. Currently, clinicians need to think about how to screen, identify, and manage people who can benefit from the expansion of antiviral treatment. The concept of an “uncertain period” or “gray area” is helpful for clinicians to implement scientific and effective individualized treatment and exemplary management for various chronic HBV-infected persons. Thus, CHB patients can truly achieve “full diagnosis and treatment,” and those with normal ALT levels, positive HBV DNA, and older chronic HBV infection can benefit from antiviral treatment as soon as possible. At present, only scientific, effective, and timely antiviral treatment can reduce the probability of HBV-related cirrhosis and liver cancer, improve patient survival rates and quality of life, and ultimately eliminate hepatitis B. The existing antiviral strategies are not ideal for the absolute prevention of HBV-related health risks. We need to develop new treatments that can clear HBV cccDNA in liver cells and eliminate the virus from the host genome, which may be more reassuring for those who initiate antiviral therapy, as long as HBV DNA is positive.

Persistent virus replication is the root cause of disease progression. Untimely treatment and inappropriate drug withdrawal may both lead to hepatitis B-related adverse events. It is necessary to expand the indications for antiviral treatment. However, if the focus were only on persuading patients to initiate antiviral treatment early, without a global perspective, and ignoring patient compliance management, it would go against the original intention of expanding antiviral treatment.

## Author contributions

PF, L-QL, and E-QC conceived this review and collected the literature. E-QC conducted the study supervision and revised the manuscript. All authors contributed to the article and approved the submitted version.

## Conflict of interest

The authors declare that the research was conducted in the absence of any commercial or financial relationships that could be construed as a potential conflict of interest.

## Publisher’s note

All claims expressed in this article are solely those of the authors and do not necessarily represent those of their affiliated organizations, or those of the publisher, the editors and the reviewers. Any product that may be evaluated in this article, or claim that may be made by its manufacturer, is not guaranteed or endorsed by the publisher.
